# Supplementary material for: Antimicrobial and anti-biofilm activity of a thiazolidinone derivative against Staphylococcus aureus in vitro and in vivo
Source: Microbiol Spectr. 2024 Feb 8;12(3):e02327-23. doi: 10.1128/spectrum.02327-23 (PMC10913468; doi:10.1128/spectrum.02327-23)

Supplementary material file: The synthetic method for TD-H2-A.

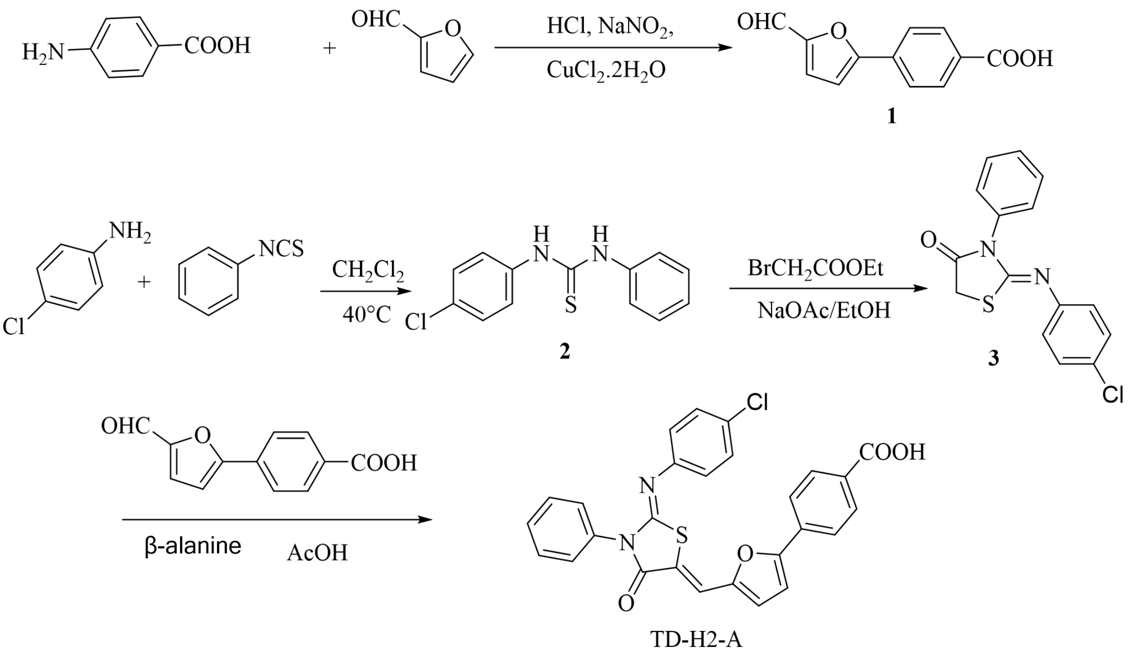

Add para aminobenzoic acid (10 mmol), water (8 mL), and concentrated hydrochloric acid (5 mL) to a 100 mL reaction flask. Then add a solution of $NaNO_2$ (12 mmol) and water (5 mL) to the system at 0°C and stir for 30 minutes. Finally, add a mixture of 2-furan formaldehyde (10 mmol) and acetone (4 mL), a solution of $CuCl_2.2H_2O$ (3 mmol) and water (2 mL), and react at room temperature for 20 hours. At the end of TLC detection, the reaction solution was filtered, washed with water, and dried to obtain compound **1** with a yield of 75%.

Dissolve 4-Chloroaniline(10mmol) in dichloromethane(15mL), add phenyl isothiocyanate(10 mmol) dropwise and stir at room temperature for 30 minutes, then raise the temperature to 40 °C, continue the reaction for 50 minutes, detect by TLC until the end, and extract with ethyl acetate (3×15 mL), combined with organic phase, dried with anhydrous magnesium sulfate, filtered, and concentrated under reduced pressure.The obtained residue was recrystallized (dichloromethane/petroleum ether=1/10) to obtain product **2**.

Add ethyl bromoacetate (6 mmol) and anhydrous sodium acetate (6 mmol) to compound **2** (5 mmol), and add solvent anhydrous ethanol (15 mL). Heat and reflux for 6 hours. TLC monitoring until the end, stop the reaction, cool to room temperature, concentrate under reduced pressure, and extract with ethyl acetate (3×30 mL), combined with organic phase, dried with anhydrous magnesium sulfate, filtered, and concentrated under reduced pressure. The obtained residue was

separated and purified using column chromatography (ethyl acetate/petroleum ether=1/10) to obtain compound **3**.

Add compound **1** (1.2 mmol) to compound **3** (1 mmol), β- Alanine(2 mmol) was added with solvent acetic acid (5 mL), heated and refluxed for 6 hours. TLC monitoring was carried out until the end. The reaction was stopped and cooled to room temperature. Solid precipitates were added to ice water, filtered, and recrystallized (methanol/dichloromethane=1/5) to obtain product TD-H2-A.

Yellow solid. yield: 0.29 g (58 %). mp 290-293 ºC. ^{1}H NMR (400 MHz, DMSO) δ 13.01 (s, 1H), 8.13 (s, 1H), 7.98 (s, 2H), 7.85 (s, 3H), 7.75 (s, 1H), 7.53 (d, *J* = 28.4 Hz, 7H), 7.06 (s, 2H).^{13}C NMR (101 MHz, DMSO) δ 167.3, 165.7, 150.8 148.4, 147.2, 138.2, 136.9, 136.4, 135.5, 131.0, 130.8, 130.0, 129.5, 129.4, 129.3, 129.0, 127.4, 126.2, 124.4, 123.2, 119.6. HRMS (ESI) *m/z*: calcd for $C_{27}H_{16}ClN_2O_4S^-$ ([M-H]$^-$) 499.9530, found 499.9528.

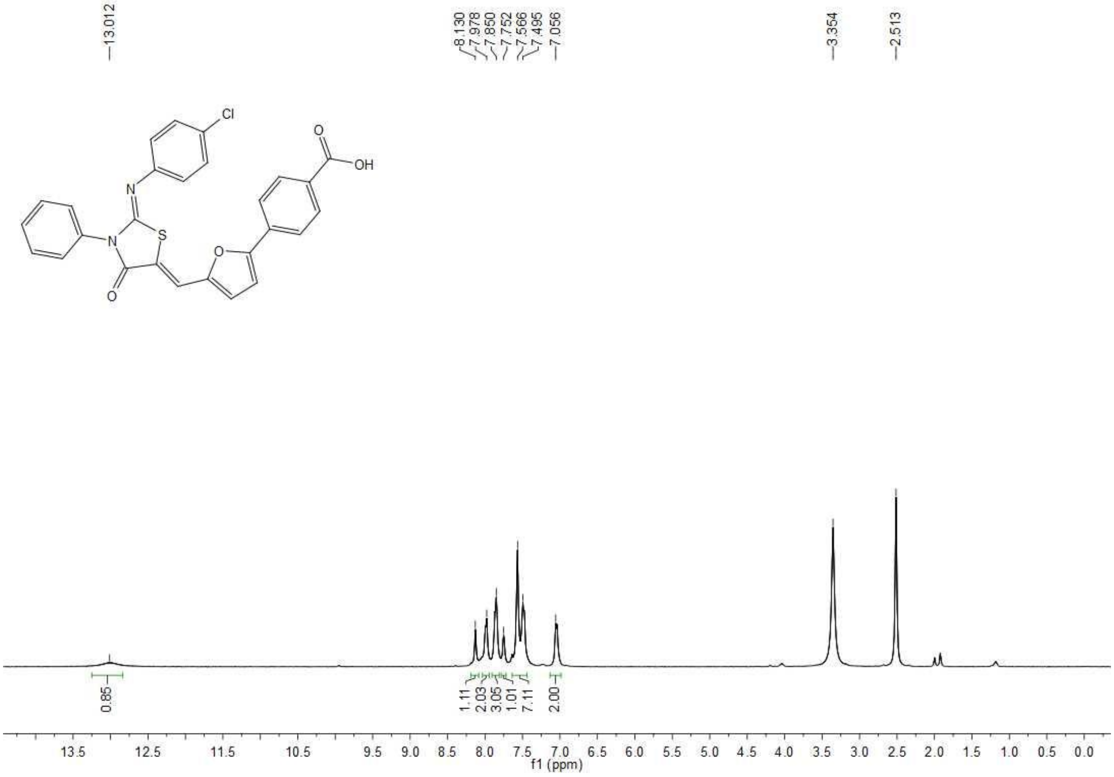

^{1}H NMR spectrum of TD-H2-A

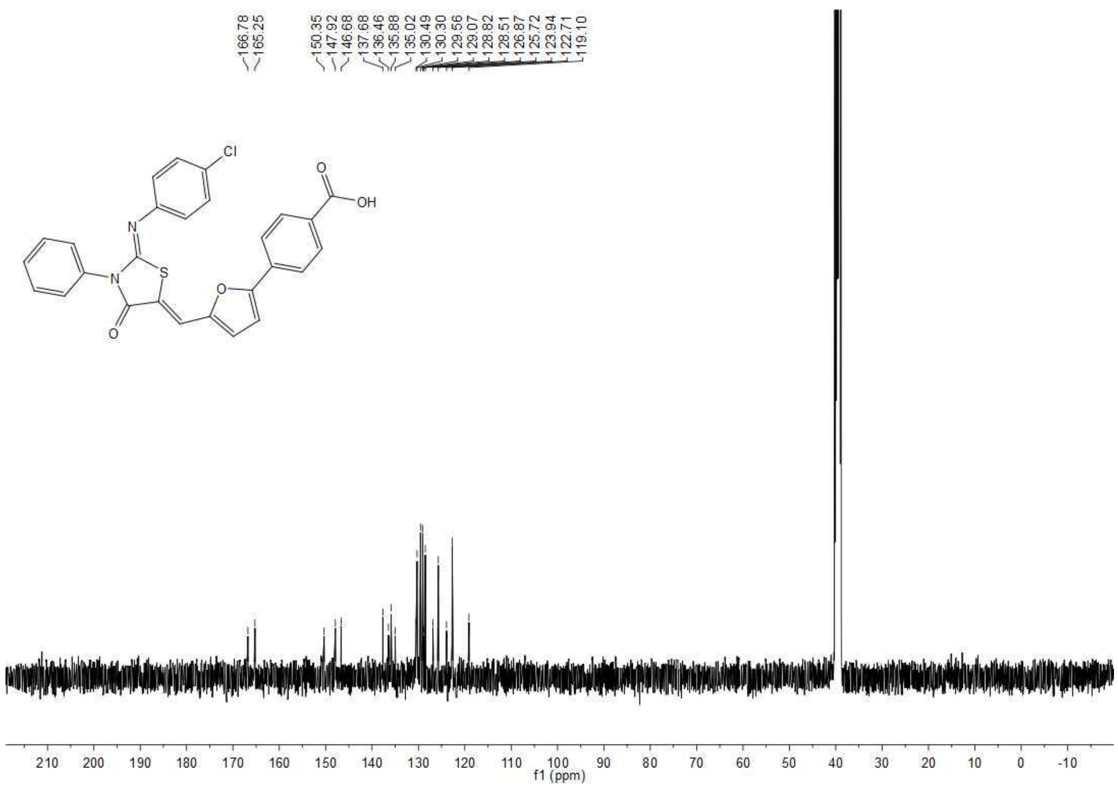

^{13}C NMR spectrum of TD-H2-A

Supplementary Table S1. Comparing the whole genome sequencing of HG001 treated with 4× MIC TD-H2-A and no treated with TD-H2-A.

CHROM	POS	TYPE	REF	ALT	GENE	PRODUCT
Gene-ID "BSR30_RS01405"	304939	snp	C	A	*BSR30_RS01405*	pseudouridine-5'-phosphate glycosidase
Gene-ID "BSR30_RS02190"	453787	ins	TAAG	TAAGAAAG	*rrf*	5S ribosomal RNA
Gene-ID "BSR30_RS04350"	849035	snp	G	A	*mnhD1*	Na+/H+ antiporter Mnh1 subunit D
Gene-ID "BSR30_RS05085"	1002603	ins	G	GAATTAACATTATTGC	*BSR30_RS05085*	TrkA family potassium uptake protein
Gene-ID "BSR30_RS07385"	1473465	ins	TAA	TGCCGCTTCAA	*BSR30_RS07385*	phage tail tape measure protein
	1473474	ins	T	TAATC		
	1473475	ins	GC	GTTGTACATAATCCC		
	1473478	ins	C	CATAATCCCACA		
	1473483	snp	T	G		
	1473484	snp	T	C		
	1473485	snp	T	C		
Gene-ID "BSR30_RS12160";	2293677	snp	A	T	*BSR30_RS12160*	hypothetical protein
	2293678	snp	T	A		
	2293679	snp	A	T		

CHROM: chromosome; POS: position; REF: reference allele; ALT: alternative allele; ins:insertion; snp:single nucleotide polymorphism.

Figure.S1 The hemolysis of TD-H2-A on healthy human erythrocytes.

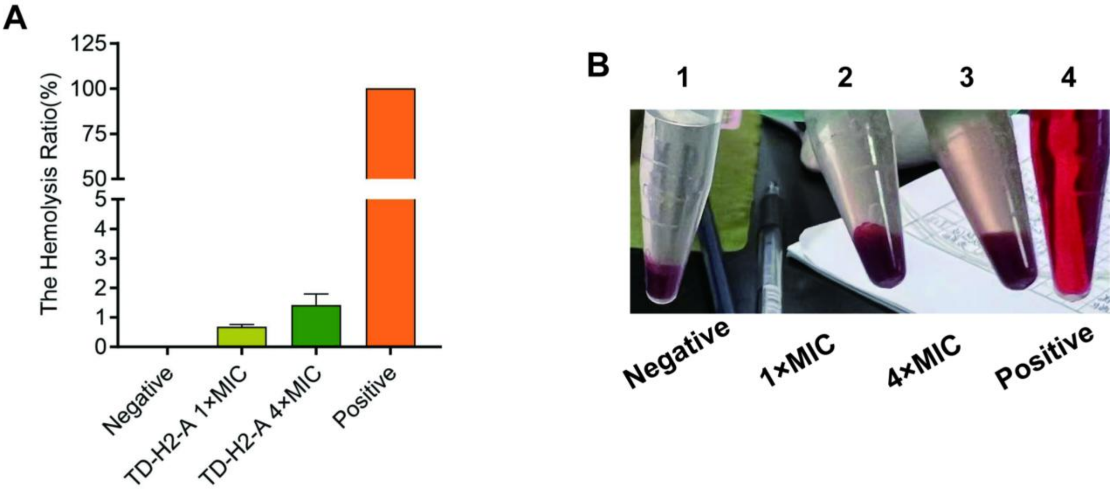

Supplement: Supplemental material — Synthetic method for TD-H2-A, Table S1, and Figure S1. [file spectrum.02327-23-s0001.pdf]
